# Improving the Solubility, Dissolution, and Bioavailability of Metronidazole via Cocrystallization with Ethyl Gallate

**DOI:** 10.3390/pharmaceutics13040546

**Published:** 2021-04-14

**Authors:** Jinhui Li, Xinghui Hao, Chenguang Wang, Haiyan Liu, Lianchao Liu, Xin He, Changquan Calvin Sun

**Affiliations:** 1 College of Veterinary Medicine, Hebei Agricultural University, Baoding 071000, China; lijh314@gmail.com (J.L.); haoxinghui107@gmail.com (X.H.); llc950730@gmail.com (L.L.); 2 Pharmaceutical Materials Science and Engineering Laboratory, Department of Pharmaceutics, College of Pharmacy, University of Minnesota, Minneapolis, MN 55455, USA; wang4889@umn.edu; 3 College of Science, Hebei Agricultural University, Baoding 071000, China; lhybdlele78@gmail.com

**Keywords:** metronidazole, ethyl gallate, cocrystal, solubility, dissolution, pharmacokinetics

## Abstract

Metronidazole (MTZ) is an antibacterial drug widely used for the treatment of protozoan and anaerobic infections in humans and animals. However, its low bioavailability necessitates the frequent administration of a high dose to attain an effective plasma concentration profile for therapy. To reduce the dose of MTZ, we have prepared a new cocrystal between MTZ and ethyl gallate (EG). The solid-state properties of MTZ-EG were characterized using complimentary techniques, including thermal, spectroscopic, microscopic, and X-ray crystallographic methods. The MTZ-EG cocrystal exhibits a higher solubility and faster dissolution than MTZ. The bioavailability of MTZ in rats was increased by 36% when MTZ-EG was used.

## 1. Introduction

**Scheme 1:**
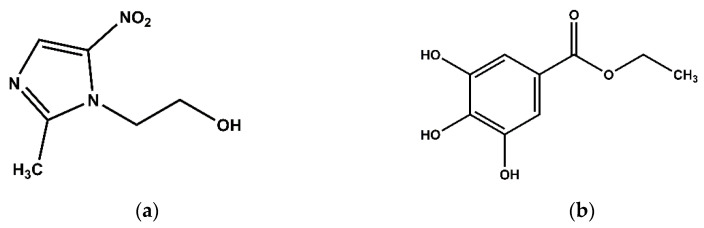
Chemical structures of (**a**) metronidazole (MTZ, MW= 171.15 g/mol) and (**b**) ethyl gallate (EG, MW= 198.17 g/mol).

Metronidazole (MTZ, Scheme 1a), [1-(2-Hydroxyethyl)-2-methyl-5-nitroimidazole], has antiprotozoal and antibacterial activities [1]. MTZ, which is on the World Health Organization’s List of Essential Medicines, has been used to treat infections caused by Gram negative anaerobic bacteria, such as *helicobacter pylori* [2]. MTZ is the first-line therapy for the treatment of patients with infections caused by *Trichomonas* (a kind of protozoan) [1,3]. It is effective in treating infectious diseases of the oral cavity and periodontitis [4,5]. The typical oral dosage of MTZ is 500 mg. However, a single dose of 2 g of MTZ may be recommended in the treatment of *Gardnerella vaginalis* infection [6]. The frequent clinic use of MTZ in large doses was thought to contribute to the pollution of soil and water and the spread of drug-resistant bacteria, which presents an increasing threat to global public health [7,8]. Additionally, some metabolites of MTZ can cause abnormalities in the nervous, respiratory, cardiovascular and immune systems [9] as well as side effects, such as peripheral neuropathy, transient neutropenia (in 7.5% of patients), and nausea (in 10% of patients) [1]. MTZ is also commonly used in cats [10], dogs [11,12], cows [13] and horses [14], despite the known genotoxic, carcinogenic and mutagenic properties of nitroimidazoles and their metabolites to mammals and humans [15,16]. To reduce the side effects in humans and animals, minimize the environmental impact and mitigate the threat of drug-resisting microbials, efforts have been made to either improve the bioavailability of MTZ [17] or synthesize new compounds with better safety profiles [18,19,20,21,22,23,24,25].

One pharmaceutical technology for enhancing the bioavailability is cocrystallization [26,27], which has also been used to improve drug physicochemical properties, such as solubility [28,29,30], dissolution rate [31,32,33], stability [34] and tabletability [35,36]. MTZ cocrystals with pyrogallol [37], 2,5-dihydroxy-benzoic acid, 2,6-dihydroxy-benzoic acid and 3,5-dihydroxy-benzoic acid [38], and gallic acid [3] have been reported. The cocrystallization of metronidazole with benzoic acid and mono-, di-, tri-hydroxybenzoic acids was prepared by thermal ink-jet printing to attain an excellent consistency in the particle size distribution [39]. However, none of these MTZ cocrystals were reported to have an enhanced solubility and bioavailability. The aim of this work was to prepare a new cocrystal of MTZ with an enhanced solubility and dissolution and to evaluate its potential application in improving the bioavailability of MTZ.

## 2. Materials and Methods

### 2.1. Materials

Metronidazole (MTZ) (purity > 99.9%) was a gift from Baoding Jizhong Pharmaceutical Co., Ltd. (Baoding, China). Ethyl gallate (EG, Scheme 1b) was obtained from Aladdin Reagent Inc. (Shanghai, China). All organic solvents used were of HPLC or analytical grade and were purchased from various commercial sources. All chemicals and solvents were used as received. Water was double-distilled.

### 2.2. Methods

#### 2.2.1. Preparation of Cocrystal

Metronidazole-ethyl gallate cocrystal (MTZ-EG) was prepared by liquid-assisted grinding. A mixture of metronidazole (25.7 mg/0.15 mmoL) and ethyl gallate (29.7 mg/0.15 mmoL) was added to a 2 mL stainless steel grinding jar. The mixture was then ground along with 20 μL methanol for 30 min at a frequency of 20 Hz using a Vibration Mill (GT300, Beijing Greatman Instrument Co., Ltd., Beijing, China).

#### 2.2.2. Preparation of Single Crystals

Single crystals of MTZ-EG were prepared by slow evaporation of a 10 mL solution of MTZ (154.2 mg, 0.9 mmoL) and EG (178.2 mg, 0.9 mmoL) in water. The solution was obtained by heating at 60 °C in a 20 mL glass vial. Then, the clear solution was allowed to cool down naturally to room temperature and was left on a laboratory bench undisrupted with the vial loosely covered to allow slow evaporation. After about one week, light yellow needle single crystals were obtained.

#### 2.2.3. Single Crystal X-ray Diffraction (SCXRD)

Single crystal X-ray diffraction data were collected at 150 K on an Agilent Technologies Gemini A Ultra system with graphite monochromated Cu Kα radiation (λ = 1.54178 Å). Data reduction and cell refinement were performed using CrysAlis^PRO^. The crystal structure was solved using Olex-2 by direct methods and refined through the full matrix least-squares method on *F^2^*. Hydrogen positions on oxygen were located in Fourier difference electron density maps. Hydrogen atoms associated with carbon atoms were refined in geometrically constrained riding positions. All non-hydrogen atoms were refined with anisotropic displacement parameters.

#### 2.2.4. Powder X-ray Diffraction (PXRD)

PXRD analysis was performed on a TD-3700 Advanced diffractometer (Dandong Tongda Science and Technology Co., Ltd., Liaoning, China) operated with Cu Κα radiation (λ = 1.54178 Å) at 30 kV and 10 mA. Data were recorded over the range of 5–35° (2*θ*) in a continuous scan mode with a step size of 0.015° (2*θ*) and a dwell time of 0.1 s.

#### 2.2.5. Fourier Transform Infrared (FT-IR) Spectroscopy

FT-IR spectra were collected under ambient condition using a Nicolet iS5 spectrometer. The spectral data ranged from 4000 to 400 cm^−1^, with a resolution of 2 cm^−1^. Approximately 1–2 mg of powder of MTZ, EG or MTZ-EG was mixed with KBr in an agate mortar, and a pellet was made on a hydraulic press at a pressure of 10 MPa for 1 min. A total of 64 scans were collected for each sample, and the average spectrum was reported.

#### 2.2.6. Differential Scanning Calorimetry (DSC) and Thermogravimetry Analysis (TGA)

DSC and TGA thermograms were recorded using an SDT Q600 simultaneous thermal analyzer (TA instrument, New Castle, DE, USA). Samples (3–5 mg) were placed in an aluminum crucible and heated at a constant rate of 10 °C/min from room temperature to 400 °C under nitrogen purge (100 mL/min).

#### 2.2.7. Scanning Electron Microscopy (SEM)

The morphological evaluation of the samples was carried out using a scanning electron microscope (S4800, Hitachi, Japan) operated at 20 kV. Samples were mounted on a copper grid with double-sided carbon tape and sputtered with gold.

#### 2.2.8. Powder Dissolution and Intrinsic Dissolution Rate (IDR)

The powder dissolution and intrinsic dissolution profiles of MTZ and MTZ-EG in water were collected using milled and sieved (75–150 μm) powders. During the powder dissolution experiments, an excess amount of solid sample was suspended in 10 mL of water in a flask and stirred at 250 rpm using an overhead stirrer at 37 °C. An aliquot of the slurry was withdrawn at predetermined time points (0, 2, 5, 10, 15, 20, 30, 45, 60, 90, 120, 180, 240, 300 and 360 min) and immediately passed through a 0.22 μm PES (polyether sulfone) filter membrane. The filtrates were diluted, and the absorbance was measured using a UV-2450 spectrophotometer (Shimadzu, Japan) at 320 nm, where EG does not interfere with the determination of the MTZ concentration. Absorbance values were converted to MTZ concentrations using a previously established calibration curve. 

The intrinsic dissolution rate (IDR) measurements were carried out using a dissolution apparatus (D-800LS, Tianjin Jingmi Instrument Co., Ltd., Tianjin, China). In each experiment, 500 mL of deionized water was equilibrated at 37 °C and stirred at 100 rpm. Approximately 300 mg of each solid was compressed in a die on a hydraulic press (1 ton for 30 s) to form a pellet with a diameter of 13-mm. The pellet was coated with paraffin wax, leaving one circular face free for dissolution. The pellet was immersed in the dissolution medium, and 1 mL of the medium was withdrawn at 2, 5, 10, 15, 20, 25, 30, 35 and 40 min. An equal volume of fresh medium was immediately replaced after each sample withdrawal. The concentration of MTZ was measured by UV/vis spectrophotometry in the same way as that for powder dissolution. The IDR was calculated from the slope of the initial linear portion of each dissolution curve. All dissolution experiments were performed in triplicate (*n* = 3).

After the powder dissolution and IDR experiments, the undissolved powders or pellets were recovered and analyzed by XRD. 

#### 2.2.9. Standard Curve for Determining Plasma Concentration of MTZ

Accurately weighed MTZ, ~10 mg, was dissolved into methanol to prepare a MTZ stock solution with a concentration of 1000 μg/mL. The plasma samples containing MTZ were prepared by adding different volumes of the MTZ standard solution into 90 μL blank rat plasma to attain concentrations of 20, 5, 1, 0.2, 0.05 and 0.025 μg/mL. After mixing, protein precipitation and separation, MTZ concentrations in these standard solutions were determined by HPLC (Model 1525, Waters Corporation, MA, USA) equipped with a PDA detector (Waters 2998 Photodiode Array Detector, MA, USA) and a C18 column with a 5 μm particle size, 4.6 mm inner diameter and 250 mm length (Waters, Ireland). The column was maintained at 37 °C. A mobile phase composed of methanol-water (30:70) was used throughout the analysis at a flow rate of 1.0 mL/min. The PDA detector was set at 320 nm. A linear regression of the MTZ peak area–concentration was performed to establish a calibration curve.

#### 2.2.10. Pharmacokinetic Study in Rats

The pharmacokinetics of MTZ and MTZ-EG were assessed in male Sprague–Dawley rats (8–9 weeks of age; 220–250 g in weight, SPF Biotechnology Co., Ltd., Beijing, China). After overnight fasting, 10 healthy rats were randomly divided into two groups before dosing (*n* = 5 in each group). The experiments were conducted in accordance with the guidelines approved by the Institutional Animal Care and Ethical Committee of the Hebei Agricultural University. Powder samples of MTZ and MTZ-EG suspended in 0.5% sodium carboxyl methyl cellulose were administered to rats using a gavage vehicle at a single dose corresponding to 50 mg/kg of MTZ. Blood samples (about 0.5 mL) were collected from the eyeball vein at 5 min, 10 min, 20 min, 30 min, 45 min, 1 h, 2 h, 3 h, 4 h, 6 h, 8 h, 12 h and 24 h after oral administration. Normal heparin was used as an anticoagulant. The blood was centrifuged at 6000 rpm for 10 min, and the plasma samples were stored at 20 °C until further analysis. To 100 μL of plasma, 300 μL of methanol was added and vortexed for 2 min and then centrifuged for 10 min at 12,000 rpm to precipitate proteins. The supernatant was filtrated by a membrane filter prior to HPLC analysis. Several key pharmacokinetic parameters, including the maximal plasma concentration (C_max_), time required to reach the C_max_ (*T*_max_), area under the plasma concentration–time curve (AUC_0–24_) and half-life (t_1/2_), were evaluated from the plasma concentration–time profile for each subject using DAS 3.5 by a noncompartmental model.

## 3. Results and Discussion

**Figure 1:**
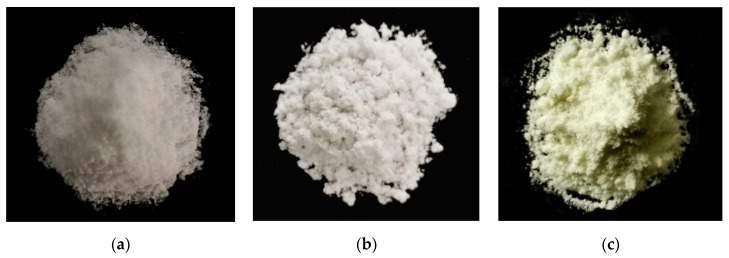
Photographs of powders of (**a**) MTZ, (**b**) EG and (**c**) MTZ-EG. The color change after the two starting powders were grinded together is evident.

The powder prepared by liquid-assisted grinding was yellow, while the starting powders were white and off-white (Figure 1). The color change is visual evidence that suggests a possible phase change upon grinding.

### 3.1. PXRD Analysis

**Figure 2:**
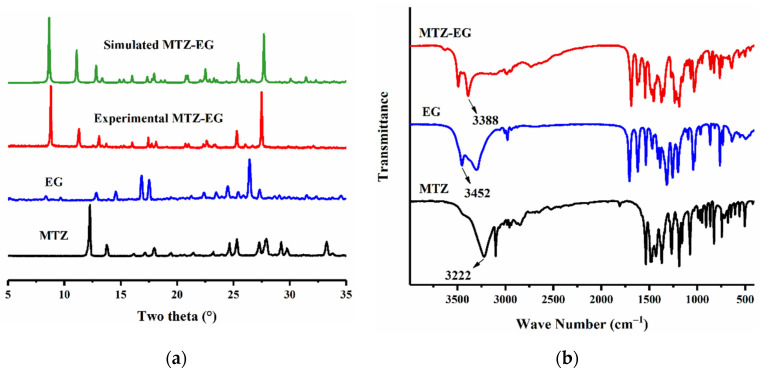
(**a**) PXRD patterns and (**b**) FT-IR spectra of MTZ-EG, MTZ and EG.

In the PXRD pattern of the grinded powder, the characteristic peaks of MTZ (12.3°, 12.7°, 17.9°) and EG (12.8°, 14.5°, 16.8°) disappeared, and new peaks (8.8°, 11.3°, 13.3°) appeared after grinding (Figure 2a), suggesting the powder was not a simple physical mixture between MTZ and EG and that a new solid phase formed. This is consistent with the observed color change (Figure 1). 

### 3.2. Crystal Structure

**Figure 3:**
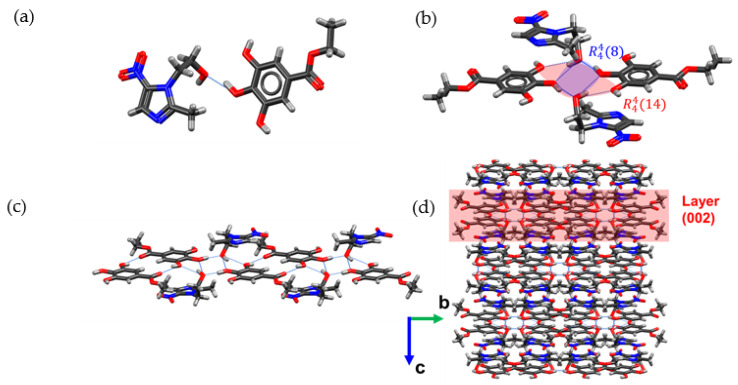
(**a**) Asymmetric unit, (**b**) hydrogen bonds between the two pairs, (**c**) 1D chain and (**d**) 3D packing of MTZ-EG viewed along the *a* axis (a 2D layer is highlighted).

The key parameters during the crystallographic data collection and refinement of MTZ-EG are summarized in Table 1. The main hydrogen bond distances and angles are provided in Table 2. MTZ-EG belongs to the monoclinic *I2/a* space group. The asymmetric unit of MTZ-EG contains one MTZ molecule and one EG molecule (Figure 3a), which are connected through an O-H^…^O (2.668 Å) hydrogen bond to form a hetero-dimer. Two hetero-dimers are further linked through O-H^…^O (2.900 and 3.037 Å) hydrogen bonds to form a hetero-tetrameric building unit. The four molecules form a stable unit through a series of hydrogen bonds, resulting in a network described by two overlapping $R_{4}^{4}\left(8\right)$ and $R_{4}^{4}\left(14\right)$ motifs (Figure 3b). Each tetramer connects to neighboring tetramers via complementary O-H^…^O (2.708 Å) hydrogen bonds on either side to form an infinite 1D chain along the *b*-axis (Figure 3c). The two MTZ molecules of each tetramer protruding on the opposite sides of the plane defined by the aromatic rings of EG. Each MTZ molecule connects to an EG molecule in the neighboring 1D chains through O-H^…^N (2.781 Å) hydrogen bonds to eventually form 2D layers along (0 0 2), which stack to form the 3D structure (Figure 3d).

The PXRD pattern of the grinded powder matched the PXRD pattern calculated from the solved crystal structure of MTZ-EG well (Figure 2a), affirming that the bulk sample prepared by liquid-assisted grinding was phase pure MTZ-EG.

### 3.3. Fourier Transform Infrared (FT-IR) Spectroscopy

FT-IR spectra can be used to infer changes in hydrogen bonding upon cocrystallization by analyzing the frequency shifts of the vibrational bands of the functional groups under consideration [40]. Since the frequency of O-H stretching generally falls in the 3600–3200 cm^−1^ spectral range, characteristic absorption peaks at 3222 cm^−1^ in the spectrum of MTZ and 3452 cm^−1^ in the spectrum of EG are assigned to O-H stretching. The spectrum of MTZ-EG has a new absorption peak at 3388 cm^−1^, indicating the participation of hydroxyl groups of MTZ and EG in new hydrogen bonding (Figure 2b).

### 3.4. Thermal Analyses

**Figure 4:**
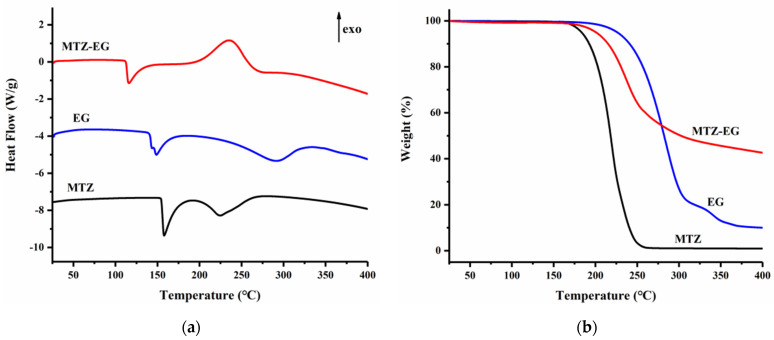
(**a**) DSC and (**b**) TG curves of MTZ-EG, MTZ and EG.

**Figure 5:**
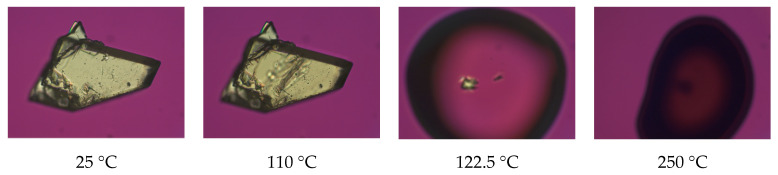
The hot-stage image of MTZ-EG at different temperatures.

The TGA and DSC thermograms of MTZ, EG and MTZ-EG are shown in Figure 4. MTZ shows an endothermic event that peaks at 158 °C, followed by a second endotherm spanning 200–275 °C (Figure 4a). The first endotherm is in agreement with the reported melting temperature of MTZ [41]. The second endotherm is attributed to the evaporation of the melt since it corresponds to the weight loss with an onset temperature of ~175 °C (Figure 4b). The thermal behaviors of EG are qualitatively similar to that of MTZ, and the same interpretation can be offered (Figure 4a,b). MTZ-EG has an endothermal event that peaks at 110 °C, which is distinctly lower than the melting temperatures of both MTZ and EG (Figure 4a). This endothermal event is positively identified as the melting of MTZ-EG crystals because the hot-stage microscopy shows the onset of MTZ-EG melting at ~122 °C (Figure 5). The exotherm in the temperature range of 190–275 °C cannot be due to evaporation, which should be endothermic. Crystallization is also excluded based on the hot-stage microscopy data. Regardless of the nature of this exothermal event, molecules in the melt of MTZ-EG interact again to form species with lower enthalpy (Figure 3b). Such species are likely hydrogen-bonded hetero-tetramers in MTZ-EG, which would be less volatile than MTZ or EG alone. This is consistent with the markedly reduced extent of weight loss at temperatures higher than 300 °C when compared to both the MTZ and EG melts (Figure 4b). 

### 3.5. Scanning Electron Microscopy (SEM)

**Figure 6:**
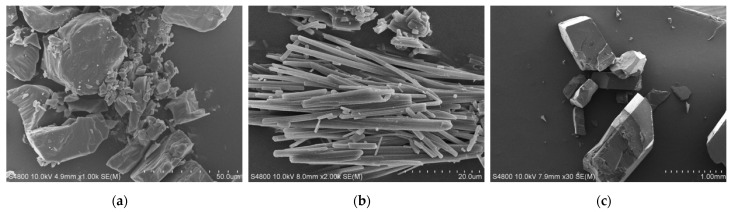
SEM images of (**a**) MTZ (×1000), (**b**) EG (×2000) and (**c**) MTZ-EG (×30).

The MTZ-EG crystals are large block-shaped (Figure 6c), which is distinct from the irregular shape of MTZ crystals (Figure 6a) and needle-shaped EG crystals (Figure 6b). 

### 3.6. Powder Dissolution and Intrinsic Dissolution Rate 

**Figure 7:**
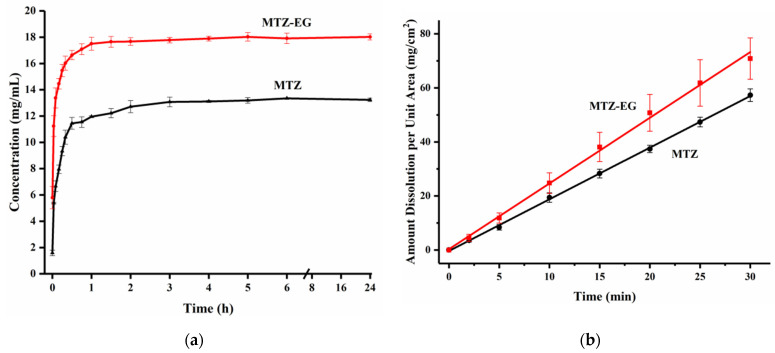
(**a**) Powder dissolution rate and (**b**) IDR of MTZ-EG and MTZ.

Compared to MTZ, the powder dissolution profile of MTZ-EG suggests that it can reach a higher plateau concentration more quickly (Figure 7a). Since the PXRD data confirmed no phase change at the end of the powder dissolution experiments of both MTZ and MTZ-EG, the plateau concentrations are the solubilities of the respective crystal forms. Thus, the apparent solubility of MTZ-EG (17.91 mg/mL) is approximately 36% higher than that of MTZ (13.19 mg/mL) in water. The IDR value of MTZ-EG (2.43 mg·cm^−2^·min^−1^) is approximately 1.27 times that of MTZ (1.91 mg·cm^−2^·min^−1^) (Figure 7b). The higher IDR of MTZ-EG is close to what is expected based on its 36% higher solubility than MTZ. Since the time of contact with the dissolution medium was much shorter (30 min) in the IDR experiments than in the powder dissolution experiment (24 h), no phase change of the pellets was detected by XRD at the end of the IDR experiment.

### 3.7. Pharmacokinetic Study

**Figure 8:**
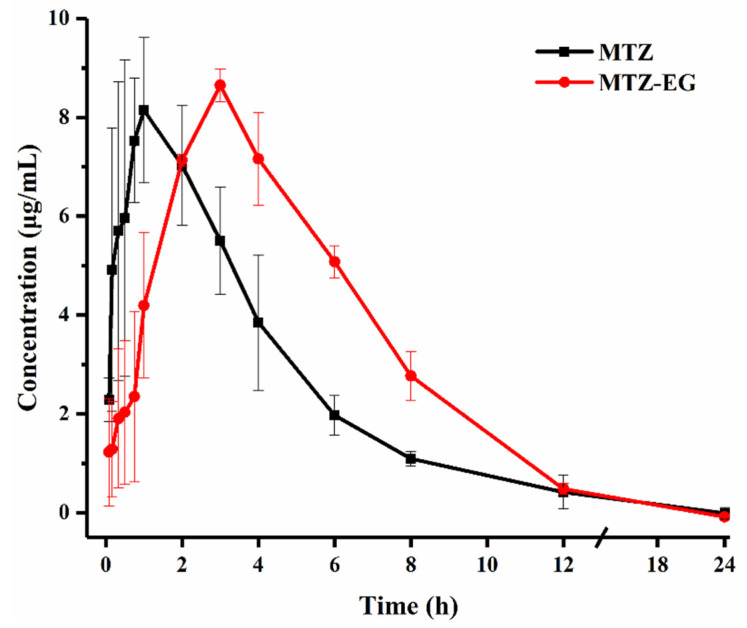
Mean plasma concentration–time profiles of the MTZ and MTZ-EG after oral administration to rats (*n* = 5).

The plasma concentration–time profiles of MTZ and MTZ-EG after oral administration in rats are shown in Figure 8. The key pharmacokinetic parameters are given in Table 3. The AUC_0~24_ of MTZ-EG (3181.84 ± 212.97 μg/mL·min) is 1.36-fold that of MTZ, which corresponds very well with the higher solubility of MTZ-EG. MTZ-EG shows a slightly higher peak plasma concentration (8.64 ± 0.33 μg/mL) than MTZ (8.15 ± 1.46 μg/mL). Meanwhile, the *T*_max_ of MTZ-EG is approximately 3.27-fold that of MTZ, which is inconsistent with the faster release of MTZ-EG observed in the powder dissolution experiment (Figure 7a). A possible explanation is that the presence of EG slows down the permeation of MTZ through the gut wall, which leads to slower absorption [42]. However, this delayed absorption does not affect bioavailability. Hence, the use of the MTZ-EG cocrystal remains a feasible strategy for reducing the dose of MTZ in oral tablets. 

The good physical stability and the improved in vivo biopharmaceutical performance of MTZ-EG make it a promising crystal form for developing a new tablet product of MTZ. However, although phase-pure MTZ-EG cocrystal powder could be prepared by the LAG method, further work is needed to explore other processes that can be used to prepare MTZ-EG in large amounts.

## 4. Conclusions

In this study, we have investigated the possibility of improving the bioavailability of MTZ by using a new cocrystal with ethyl gallate. Compared to MTZ, the MTZ-EG cocrystal exhibits a higher solubility, faster dissolution and higher bioavailability. Additionally, MTZ-EG is physically stable in water for at least 24 h. Overall, the improved solubility and solid-state stability during dissolution make MTZ-EG a suitable candidate for further development into an oral solid dosage form with an improved biopharmaceutical performance.

## Figures and Tables

**Table 1:** Crystallographic data and refinement parameters for MTZ-EG.

Formula	C_15_H_19_N_3_O_8_
CCDC no.	2,063,084
Formula weight	369.33
T/K	200
Wavelength/Å	1.54184
Crystal system	Monoclinic
Space group	I 2/a
*a*/Å	16.5805 (7)
*b*/Å	13.2924 (4)
*c/*Å	16.5248 (6)
*α/*°	90
*β/*°	106.018 (4)
*γ/*°	90
V/Å^3^	3500.6 (2)
Z	8
D_x/_g cm^−3^	1.402
R_int_	0.048162
T_min_	0.536
T_max_	1
h, k, l_max_	21, 16, 21
Mu (mm^−1^)	0.985
F000	1552
θ_max_	79.654
GooF	1.056
R_1_, wR_2_ [I ≥ 2σ(I)]^a^	0.0878, 0.2616
R_1_,wR_2_ [all data]^b^	0.0947, 0.2648

^a^ R_1_ = Σ‖F_o_| − |Fc‖/Σ|F_o_|·^b^ wR_2_ = [Σ[w(F_o_^2^ − Fc^2^)^2^]/Σw(F_o_^2^)^2^]^1/2^, w = 1/[σ^2^ (F_o_) ^2^ + (aP) ^2^ + bP], where P = [(F_o_^2^) + 2F_c_^2^ ]/3.

**Table 2:** Hydrogen bond distances and angles in MTZ-EG.

D-H···A	D-H (Å)	H···A (Å)	D···A (Å)	D-H···A (°)
O6-H6···O7 ^i^	0.82	1.92	2.708 (3)	161.5
O5-H5···N2 ^ii^	0.82	1.96	2.781 (3)	178.1
O3-H3···O4 ^iii^	0.82	2.38	3.037 (2)	137.8

Symmetry codes: (i) −x + 1, −y + 2,-z + 1 (ii) x − 1/2, −y + 1, z (iii) −x + 1, −y + 1, −z + 1.

**Table 3:** Summary of the PK data of MTZ and MTZ-EG in rats.

	T_1/2_ (min)	T_max_ (min)	C_max_ (μg/mL)	AUC_0–24_ (μg/mL·min)
MTZ	106.95 ± 7.29	55.00 ± 8.66	8.15 ± 1.46	2345.98 ± 409.63
MTZ-EG	100.82 ± 15.98	180.00 ± 0.00	8.64 ± 0.33	3181.84 ± 212.97

## Data Availability

No new data were created or analyzed in this study. Data sharing is not applicable to this article.
